# A Promising Antibacterial Inhibition of Propolis Extracts Against Carbapenem‐Resistant Bacteria Isolated From Clinical Samples

**DOI:** 10.1002/cbdv.202500158

**Published:** 2025-08-30

**Authors:** Ülkü Zeynep Esertaş, Neşe Inal, Zehra Can, Sevgi Kolaylı

**Affiliations:** ^1^ Department of Medical Microbiology, Faculty of Medicine Ağrı İbrahim Çeçen University Ağrı Türkiye; ^2^ Department of Emergency Aid and Disaster Management, Faculty of Applied Sciences Bayburt University Bayburt Türkiye; ^3^ Department of biochemistry, Faculty of Science Kararadeniz Technical University Trabzon Türkiye

**Keywords:** carbapenem‐resistant bacteria, clinical isolated, propolis

## Abstract

The increasing inadequacy of antibiotics against multidrug‐resistant pathogens is a growing concern. In this study, the antibacterial activity of propolis extracts with different phenolic profiles against carbapenem‐resistant clinical isolates was investigated. The antimicrobial activity of propolis extract was tested against *Acinetobacter baumannii* (K17, K16, and K21), *Klebsiella pneumoniae* (K22, and K19), and *Stenotrophomonas maltophilia* (E5, E7, and E4) bacterial strains isolated from tracheal aspirate and blood cultures. The total phenolic content and phenolic composition of the extracts were analyzed. Antimicrobial activity values examined according to the disk diffusion method were measured as minimum inhibitory concentration (MIC) and minimum bactericidal concentration (MBC). The results showed that all propolis extracts showed different inhibition values against bacterial strains. As a result, the high inhibitory potential of the propolis extracts against resistant bacteria such as *A. baumannii*, *K. pneumoniae*, and *S. maltophilia* is promising for combating infections. However, the study needs to be supported by further randomized clinical studies.

## Graphical abstract

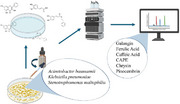

## 1 Introduction

Antibiotic resistance is a growing global health concern that threatens the effectiveness of life‐saving medications. The overuse and misuse of antibiotics, both in medical treatment and agriculture, contribute to the emergence of resistant strains of bacteria [1]. The rapid ability of bacteria to adapt and evolve makes combating antibiotic resistance particularly challenging [2]. When antibiotics are not used judiciously, bacteria can survive and continue to multiply, leading to the development of strains that are no longer responsive to standard antibiotic treatments. Various preventive measures are being developed to reduce antibiotic resistance. One of these is to produce traditional and alternative solutions in the fight against infections. The use of natural products with high antimicrobial value in this field is not new, and traditional products have been used since ancient times. Natural products such as thyme, mint, garlic, onion, ginger, tea tree oil, honey, and propolis are some of the products with high antimicrobial value [2, 3, 4].

Propolis is a resin obtained from beehives. Honeybees transform the resins they collect from nature into propolis to protect their hives from diseases and natural conditions. Propolis plays an important role in the insulation of the beehive and in the mummification of dead bees and insects. In addition, propolis plays an important role in the defense of the hive against disease agents such as bacteria and viruses. Propolis collected from the hive as raw propolis is extracted with solvents of different polarities and consumed as propolis extract [5]. The composition of raw propolis varies depending on the plant flora. Raw propolis consists of three different fractions: balsamic fraction, essential oils, and waxes.

The composition of propolis extracts varies depending on the type of solvent used, extraction method and ratio of propolis/solvent [5]. There is no single method to obtain propolis extracts from raw propolis. The content of propolis varies depending on the different methods developed by companies producing commercial propolis. However, all studies have revealed that the most ideal solvent for propolis is 70% ethanol. However, some polyalcohols such as polyethylene glycol (PEG), polypropylene glycol (PPG), monoethylene glycol (MPG), and glycerol are also used in extraction [6, 7].

Propolis is a significant natural food supplement with a wide range of applications and plays a key role in alternative and complementary medicine [8]. Propolis extracts, rich in polyphenolic compounds, are widely recognized for their potent antimicrobial, antioxidant, antiviral, anti‐inflammatory, and antitumor properties [9]. Numerous studies have demonstrated its broad‐spectrum antibacterial activity, positioning it as a viable alternative to synthetic antibiotics [3, 6]. This study aimed to evaluate the inhibitory effects of various propolis extracts against infectious bacteria isolated from clinical strains.

Therefore, this study aimed to investigate the inhibitory effects of propolis extracts, prepared using different extraction methods, against selected antibiotic‐resistant bacterial strains. Furthermore, the correlation between the phenolic composition of the extracts and their antibacterial efficacy was assessed.

## 2 Results and Discussion

In this study, the antimicrobial activities of five different propolis extracts on clinical strains were tested. The compositions of ethanolic propolis extracts prepared in different formulations are summarized in Table 1. It was determined that the total phenolic content (TPC) values of the propolis extracts varied between 27.34 and 98.34 mg GAE/mL (Table 2). It was determined that the highest TPC value and the highest TPC value was found in the 30% commercial propolis sample (P4). It is stated on the label that this sample is a 30% ethanolic Anatolian propolis. P3 and P4 propolis extracts are samples of the same commercial company, but their solvents and percentages are different. P3 propolis is 15% and dissolved in 30% PPG solvent instead of ethanol. The total phenolic substance amount of this extract, called water‐based propolis, was found to be slightly lower in ethanolic samples. Among the samples studied, the lowest amounts of phenolic substances were determined in sample P5. This commercial propolis extract is produced as a nasal spray and cannot be taken orally. The most abundant bioactive components in propolis are polyphenols, and phenolic acids are divided into two main classes: flavonoid [6, 10]. The results indicated that the total flavonoid substance amounts varied between 0.23 and 14.76 mg QE/mL. The sample containing the highest flavonoids was P4 commercial propolis, while the lowest was P5. It was determined that P1 and P2 samples had similar flavonoid content.

**TABLE 1:** Propolis extracts were used in the study.

		Preparation	Concentration (mg dry matter/mL)
P1	Raw propolis/Yıgılca/Turkiye	8 g/30 mL, 70% ethanol	260
P2	Raw propolis/mixed Anatolia	8.0 g/40 mL, 70% ethanol	200
P3	Water soluble/BEE'O	Glycol 15%	150
P4	Ethanolic extract/BEE'O	30%	300
P5	Spray with propolis/BEE'O	—	—

**TABLE 2:** Total phenolic and flavonoid contents of the propolis sample.

	P1	P2	P3	P4	P5
Total phenolic content (mg GAE/mL)	36.11 ± 1.58^a^	32.86 ± 1.49^b^	30.67 ± 0.18^a^	98.34 ± 3.79^c^	27.34 ± 1.19^d^
Total flavonoid content (mg QE/mL)	7.03 ± 0.32^a^	6.15 ± 0.62^a^	10.84 ± 1.10^b^	14.76 ± 0.58^c^	0.23 ± 0.01^d^
Total antioxidant capacity (FRAP) (µmol FeSO_4_·7H_2_O/mL)	291 ± 3.10^a^	296.00 ± 6.20^a^	552.18 ± 9.04^c^	819.30 ± 8.21^b d^	120.33 ± 5.08^e^

*Note*: Different letters (a–d) in the same columns are significantly different at the 5% level (*p* < 0.05).

Antioxidant capacities of the samples were determined according to the FRAP method, and in this method, high FRAP value indicates high antioxidant capacity. The FRAP value varied between 120.33 and 819.30 µmol FeSO_4_·7H_2_O/mL, and the highest FRAP value was found in the P5 sample. The essence of the FRAP method shows the ability of antioxidant substances contained in propolis extracts to reduce the Fe(III)–TPTZ complex [11]. Any substance that reduces oxidation or prevents its formation is called antioxidant. Different antioxidant activity methods such as radical scavenging activity and metal ion chelating activity have been described in the literature. The basis of the antioxidant test method here (FRAP) is the reduction of the Fe (III)–TPTZ complex of the antioxidant substance in the propolis extract to the Fe(II)–TPTZ complex^.^[11] As seen from the results, samples containing high phenolic substances were found to have high antioxidant capacity. The results showed that the antioxidant capacities of the propolis extract vary depending on the propolis: solvent ratio used in the preparation of the extract, the type of solvent used and the botanical properties of raw propolis. As a matter of fact, studies in literature support our findings [5, 12].

Phenolic component analysis of propolis extracts by HPLC are summarized in Table 3. Twelve phenolic acids and 12 flavonoids were analyzed according to the standard validated method [5]. While caffeic acid, CAPE, and ferulic acid were detected as the common phenolic acid in all the extracts, the amounts of gallic acid, chlorogenic acid, syringic acid, and ellagic acid were below the detection limits. caffeic acid and CAPE are polyphenols with high biological active value and propolis is one of the marker compounds [13]. While the highest amount of caffeic acid was found in the P1 sample, the highest CAPE was detected in the P2 sample. *p‐*Coumaric acid is one of the important phenolic acids found in propolis and was detected highest in the P4 sample [14]. Galangin, chrysin, and pinocembrin, which are flavonoids class compounds, were detected at major levels in all samples (Table 3). Quercetin and apigenin were detected in low concentrations, but common components were detected in all samples. Among the samples studied, P5 was found to have the lowest amounts of phenolic substances. This sample was already the sample with the lowest TPC amount and antioxidant value. Since the concentration of raw propolis dissolved in this commercial product in the spray form is low (6%), it was expected to be lower in phenolic composition than other samples.

**TABLE 3:** Phenolic composition of the propolis extracts using RP‐HPLC‐PDA.

	µg phenolic/mL extract	P1	P2	P3	P4	P5
Phenolic acids	Gallic acid	—	—	—	—	—
	Protocatechuic acid	—	—	—	0.01	0.74
	Chlorogenic acid	—	—	—	—	—
	*p‐*OH benzoic acid	—	—	—	—	—
	Caffeic acid	5.44	1.10	0,50	0.82	0.81
	Vanillic acid	—	—	—	—	—
	Syringic acid	—	—	—	—	—
	Ferulic acid	3.03	6.370	0.03	4.73	1.13
	*t*‐Cinnamic acid	5.38	—	0.78	—	—
	*p*‐Coumaric acid	1.06	—	0.90	1.43	—
	Ellagic acid	—	—	—	—	—
	CAPE	10.88	15.80	5.42	6.40	2.55
Flavonoids	Rutin	0.51	—	—	0.50	—
	Myricetin	—	—	—	—	—
	Daidzein	—	—	—	—	—
	Galangin	17.55	34.90	—	14.53	7.31
	Luteolin	—	—	—	—	—
	Quercetin	0.91	1.53	0.44	1.20	—
	Naringenin	—	—	—	—	—
	Apigenin	0.34	2.22	1.29	1.28	—
	Hesperetin	0.30	—	6.37	7.22	—
	Rhamnetin	—	—	—	—	—
	Chyrisin	23.48	20.98	11.49	14.83	—
	Pinocembrin	22.46	37.46	13.14	17.00	1.05

The study was conducted on various clinical samples. Clinical strains were isolated from blood, sputum, tracheal, and urine cultures. Antibiotic susceptibility tests of each microorganism were given in Tables 4, 5, 6. *Klebsiella pneumoniae* strains were found to be resistant to ampicillin, amoxicillin–clavulanic acid, piperacillin–tazobactam, cefuroxime, cefoxitin, ceftazidime, ceftriaxone, cefepime, ertapenem imipenem, meropenem, and trimethoprim–sulfamethoxazole. Amikasin was found to be susceptible to *K. pneumoniae* strains. *A. baumanni* strains were found to be resistant imipenem, meropenem, piperacillin–tazobactam, ciprofloxacin, trimethoprim–sulfamethoxazole. Two of the three *Acinetobacter baumanni* strains were found to be sensitive to amikacin. Three *Acinetobacter baumanni* strains were found to be susceptible increased exposure to tigecycline. Three *Stenotrophomonas maltophilia* isolates were found to be sensitive to levofloxacin and susceptible increased exposure to trimethoprim–sulfamethoxazole. The World Health Organization has published a list of priority antibiotic‐resistant pathogens that pose the greatest threat to human health. Carbapenem‐resistant *Acinetobacter baumannii* and carbapenem‐resistant and extended spectrum β‐lactamases (ESBL) producing *Enterobacteriales* are in the critical priority group for new drug development [15]. Therefore, the investigation of the potential activity of the propolis extract against multidrug‐resistant strains of *K. pneumoniae* and *A. baumannii* is of significant interest*. S. maltophilia* is an opportunistic pathogen that results in nosocomial infections in immunocompromised individuals. Treatment options to treat this pathogen are limited. Due to the adaptive nature of the intrinsically resistant mechanism towards the number of antibiotics and its ability to acquire new resistance via mutation and horizontal gene transfer, it is quite difficult medical therapy against *S. maltophilia*.

**TABLE 4:** The antibiotic susceptibility testing of microorganisms.

Microorganism	Sample	Antibiotic susceptibility (µg/mL)						
		IPM	MEM	TZP	AK	CIP	TGC	SXT
K‐17 *Acinetobacter baumannii*	Blood	≥16 R	≥16 R	≥128 R	≥64 R	≥4 R	4 I	≥320 R
K‐21 *A. baumannii*	Tracheal aspirate	≥16 R	≥16 R	≥128 R	≤2 S	≥4 R	4 I	≥320 R
K‐16 *A. baumannii*	Tracheal aspirate	≥16 R	≥16 R	≥128 R	≤2 S	≥4 R	4 I	≥320 R

Abbreviations: AK, amikacin; CIP, ciprofloxacin; I, sensitive to increased dose; IPM, imipenem; MEM, meropenem; R, resistant; S, sensitive; SXT, trimethoprim–sulfamethoxazole; TGC, tigecycline; TZP, piperacillin–tazobactam.

**TABLE 5:** The antibiotic susceptibility testing of microorganisms.

Microorganism	Sample	Antibiotic susceptibility (µg/mL)												
		AM	AMC	TZP	CXM	FOX	CAZ	CRO	FEP	ETP	IPM	MEM	AK	SXT
K‐22 *Klebsiella pneumoniae*	Tracheal aspirate	≥32 R	≥64 R	≥128 R	≥64 R	≥64 R	≥32 R	≥64 R	≥32 R	≥8 R	8 R	≥16 R	4 S	≥320 R
K‐19 *K. pneumoniae*	Urine	≥32 R	≥64 R	≥128 R	≥64 R	≥64 R	≥32 R	≥64 R	≥32 R	≥8 R	≥16R	≥16 R	4 S	≥320 R

Abbreviations: AK, amikasin; AM, ampicillin; AMC, amoxicillin–clavulanic acid; CAZ, ceftazidime; CRO, ceftriaxone; CXM, cefuroxime, cefoxitin; ETP, ertapenem; FEP, cefepime; IPM, imipenem; MEM, meropenem; SXT, trimethoprim–sulfamethoxazole; TZP, piperacillin–tazobactam.

**TABLE 6:** The antibiotic susceptibility testing of microorganisms.

Microorganism	Sample	Antibiotic susceptibility (µg/mL)	
		SXT	LEV^a^
E‐5 *Stenotrophomonas maltophilia*	Tracheal aspirate	≤20 I	0.125 S
E‐7 *S. maltophilia*	Sputum	≤20 I	0.15 S
E‐4 *S. maltophilia*	Blood	≤20 I	0.19 S

Abbreviations: I, sensitive to increased dose; LEV, levofloxacin; R, resistant; S, sensitive; SXT, trimethoprim–sulfamethoxazole.

^a^ Only the breakpoint of levofloxacin was evaluated according to the CLSI M100 (2023).

The antimicrobial activities of the propolis extracts used in our study were tested on eight different clinical strains, the antimicrobial activity values are given in terms of zone diameter as mm. The inhibitions were studied in bacterial species isolated from different patients. Table 6 gives the zone sizes measured against these bacteria. After the intravenous blood of the patients were inoculated with agar medium, bacteria were identified by the vitex method. Three different bacterial species of *A. baumannii*, *K. pneumoniae*, and *S. maltophilia* were identified in the media. It was determined that the five different propolis extracts studied showed different inhibitions. However, the highest activity was determined in P4 and P5 samples.

P1 and P2 extracts were found to have similar activity. While P1 extract showed no inhibition against the first two *A. baumannii* strains, it showed moderate inhibition against the third strain. It is thought that the antimicrobial activity of propolis is due to polyphenols. As a matter of fact, studies have reported that extracts with high phenolic composition have high antimicrobial activity [4].

It was determined that the highest antimicrobial activity among the propolis extracts other than the spray form (P5) belonged to the P4 and P3 samples. It was found surprising that although the amount of Phenolic substance in the P5 spray form sample was low, it showed high antimicrobial activity. It is thought that the high antimicrobial activity of this sample, which is used as a throat spray, is due to the honey and menthol in its composition.

In the study, two antimicrobial effects, bacteriostatic and bactericidal, were examined, and the minimum inhibition concentration (MIC) and minimum bactericidal concentration (MBC) values showing these two effects were summarized in Table 7. Bacteriostatic effect refers to the ability to stop or inhibit the proliferation of microorganisms. These substances interrupt the metabolism or reproduction processes of bacteria, pausing their proliferation, but they do not kill the bacteria [2, 16]. While the bacteriostatic effect is expressed by MIC values, MIC values were found to vary between 125 and 500 µg/mL. As can be seen from the table of MIC values, P3, P4, and P5 propolis extracts appear to be effective on the relevant bacteria.

**TABLE 7:** The minimum inhibition concentration (MIC) and minimum bactericidal concentration (MBC) (µg/mL).

Clinical isolated strains	P1		P2		P3		P4		P5	
	MIC	MBC	MIC	MBC	MIC	MBC	MIC	MBC	MIC	MBC
K‐17 *Acinetobacter baumannii*	—		125	250	−/+		250	500	−/+	
K‐21 *A. baumannii*	−/+		−/+		250	500	250	500	250	500
K‐16 *A. baumannii*	500	1000	250	500	250	500	−/+		−/+	
K‐22 *Klebsiella pneumoniae*	500	1000	500	1000	250	500	250	500	125	250
K‐19 *K. pneumoniae*	—		—		500	1000	—		250	500
E‐5 *Stenotrophomonas maltophilia*	—		−/+		500	1000	250	500	250	500
E‐7 *S. maltophilia*	−/+		−/+		250	500	500	1000	500	1000
E‐4 *S. maltophilia*	500	1000	500	1000	125	250	500	1000	250	500

Bactericidal effect refers to the ability to kill or destroy microorganisms. These substances cause the death of bacteria by damaging the cellular structure of microorganisms or blocking their basic biological processes [17, 18]. While the MBC value indicates bactericidal activity, these values were found to vary between 500 and 1000 µg/mL.

It has been shown that propolis extracts provide high inhibitory activity against many bacteria that cause infection in humans [19, 20, 21]. The bacterial growth‐inhibiting impacts of propolis have been extensively studied. Several studies have shown the efficiency of propolis as an antimicrobial agent inhibiting the growth of Gram‐positive and Gram‐negative bacteria. In literature, Saddiq and Danial examined the antimicrobial, antioxidant and probiotic activity of commercial Saudi Arabian propolis, performing antimicrobial activity tests on the pathogenic *K. pneumoniae, Pseudomonas aeruginosa, Escherichia coli, Aspergillus niger*, and *Aspergillus flavus*. The best antibacterial effect was observed with *K. pneumoniae* [22]. Stepanovic et al. [18] investigated the antimicrobial properties of 13 ethanol propolis extract samples from different regions of Serbia against 39 microorganisms, including 14 resistant or multiresistant to antibiotics, to determine synergistic activity between antimicrobials and propolis. They found that ethanol propolis extracts, irrespective of microbial resistance to antibiotics, showed significant antimicrobial activities against Gram‐positive bacteria, while Gram‐negative bacteria were less susceptible [18]. Brazilian green propolis aqueous extract was found that fast and effective against multidrug‐resistant strains of *K. pneumoniae* and *P. aeruginosa* in planktonic cultures and biofilms [23]. In our study, the highest activity was determined in P4 and P5 extract.

The analysis of the average MIC values for propolis extracts confirmed their higher efficacy against Gram‐positive than Gram‐negative bacteria. The MIC values for ethanolic extract from propolis were 117–1840 µg/mL for the Gram‐positive bacteria and 34–5000 µg/mL for the Gram‐negative bacteria [24]. In the study in which the antimicrobial activity of European propolis collected from various geographical origins was examined, the MIC values of *A. baumannii* ATCC BAA‐747 strain were found to be 5000 µg/mL [25]. According to the activity of ethanol propolis extracts, MIC values of *Klebsiella* spp. were found to be 32–3330 µg/mL [24]. In our study, MIC values were found to differ between 125 and 500 µg/mL. As can be seen from the table of MIC values, P3, P4, and P5 propolis extracts appear to be effective on the multidrug resistant clinical strain. MBC values were found to vary between 500 and 1000 µg/mL.

The antimicrobial and anti‐virulence potentials of green and red propolis from Brazil against multidrug‐resistant bacteria (MDRB) were investigated and red propolis extracts were shown to exhibit potent antimicrobial activity against Gram‐positive MDRB [26]. In a study comparable to ours, conducted on various Palestinian propolis samples, it was demonstrated that propolis extracts exhibited significant inhibitory activity against MDRB, with a particular efficacy observed against Gram‐positive bacteria [27]. In a study conducted on MDRB such as *Streptococcus pyogenes*, *Staphylococcus aureus* (MRSA), and *Enterococcus faecium* (VRE), the antimicrobial activities of propolis extracts were evaluated by determining the MICs and minimum microbicidal concentrations (MMCs). The results demonstrated that the extracts exhibited high efficacy against these bacterial strains [28].

The aim of this study was to investigate the potential inhibitory effects of various propolis extracts against MDRB isolated from clinical strains and to provide preliminary data for future research. The results indicate that propolis extracts, at varying concentrations and compositions, exhibit different levels of activity against the targeted bacterial strains. However, the specific mechanisms underlying these effects remain an area for further investigation.

## 3 Conclusions

In conclusion, all propolis extracts evaluated in this study exhibited varying degrees of inhibitory activity against carbapenem‐resistant bacteria, and it was determined that their antimicrobial effects differed based on the chemical composition of the extracts. These findings suggest that propolis extracts possess a broad spectrum of antimicrobial activity. Although the results are promising, the efficacy of propolis against infections caused by *A. baumannii*, *K. pneumoniae*, and *S. maltophilia* pathogens resistant to standard therapies requires further investigation using a larger number of samples and bacterial strains. Moreover, additional well‐controlled studies are necessary to assess the potential synergistic effects between propolis and conventional antimicrobial agents in clinical settings.

## 4 Experimental Section

### 4.1 Samples

Two propolis extracts used in the study were obtained from raw propolis in the laboratory, the remaining three extracts were purchased from the pharmacy. The propolis extracts and concentrations are summarized in Table 1. Five different propolis extracts were evaluated in this study, two of which were prepared from raw propolis. As presented in Table 1, the raw propolis was first ground into a fine powder and then extracted using 70% ethanol. The extraction process involved sonication in an ultrasonic bath for 2 h, followed by continuous shaking for 24 h. The resulting extract was filtered and subsequently used in the analyses [5].

### 4.2 Chemical Analysis of Propolis Extracts

#### 4.2.1 TPC

The Folin–Ciocalteu's assay was used to measure TPC values in the propolis extracts [29]. In a nutshell, 20 µL of the extract was combined with 400 µL of 0.2 N Folin–Ciocalteu's reagent and then diluted to 680 µL with distilled water. Following a 3‐min incubation, 400 mL of Na_2_CO_3_ (10%) was introduced, and the mixture underwent an additional 2‐h incubation at room temperature. After incubation, the absorbance was measured at 760 nm using a spectrophotometer (Thermo Scientific Evolution TM 201, UV‐VIS Spectrophotometer, USA). The TPC of the samples was determined in milligrams of gallic acid equivalents (GAE) per milliliter of the sample, employing gallic acid standards (ranging from 0.032 to 1.0 mg GAE/mL), as the line equation.

#### 4.2.2 Total Flavonoid Content

The propolis extracts were assessed following the procedure outlined by Fukumoto and Mazza [30]. In summary, 250 µL of the extract was combined with 50 µL of 10% Al(NO_3_)_3_ and 50 µL of 1.0 M NH_4_·CH_3_COO. The resultant mixture was then diluted to 3.0 mL using methanol (99%) and allowed to incubate at 25°C for 40 min, after which the absorbance was gauged at 415 nm. Total flavonoid content (TFC) was quantified in terms of mg quercetin equivalent (QUE)/g sample utilizing quercetin standards (0.031–0.5 mg QE/mL), employing the linear equation.

### 4.3 Determination of Antioxidant Capacity

The total antioxidant capacity of the samples was determined through the ferric‐reducing antioxidant power (FRAP) assay method [31]. The FRAP reagent was freshly prepared by combining 300 mM pH 3.6 acetate buffer, 10 mM 2,4,6‐tris(2‐pyridyl)‐S‐triazine (TPTZ), and 20 mM FeCl_3_ solutions in a ratio of 10:1:1, respectively. A mixture of 3 mL of the FRAP reagent and 100 µL of the sample was incubated for 4 min at 37°C, followed by measuring the absorbance at 593 nm. Standard curve preparation utilized different concentrations of FeSO_4_·7H_2_O (ranging from 1000 to 31.25 µmol/mL). The results were expressed as µmol FeSO_4_·7H_2_O equivalents per gram of the sample, utilizing the curve with the line equation.

### 4.4 DPPH Radical Scavenging Activity (SC_50_)

The assessment of free radical scavenging activity in the samples followed the protocol outlined by Molyneux [32]. In summary, 750 µL of the sample extract was combined with 750 µL of a DPPH radical solution. This mixture was then incubated in the dark for 40 min at 25°C, and the absorbance was recorded at 517 nm. The result was expressed as SC_50_, representing the sample concentration causing a 50% reduction in the DPPH• radical concentration. Lower SC_50_ values indicate higher efficacy in radical scavenging activity.

### 4.5 Phenolic Composition Analyses With RP‐HPLC‐PDA

The phenolic composition analysis of the samples employed a reverse‐phase high‐performance liquid chromatographic method (RP‐HPLC) with photodiode array detection (PDA), utilizing 25 phenolic standards. The RP‐HPLC‐PDA method's validation parameters were in line with those utilized in a previous study [5]. Before analysis, both propolis extracts underwent liquid–liquid extraction for enrichment. The ethanolic extracts were subsequently evaporated (IKA‐Werke, Staufen, Germany), and the residue was reconstituted in distilled water at pH 2. The aqueous extracts' pH was adjusted to 2 with diluted HCl. Liquid–liquid extraction involved sequential use of 5 × 3 mL diethyl ether and ethyl acetate. The collected organic phase was evaporated, and the residue was dissolved in methanol for analysis using the HPLC system (Shimadzu Corporation, LC 20AT). The results were quantified in µg/mL.

### 4.6 Clinical Strains

In the study, clinical samples were selected from strains that were sent to the microbiology laboratory of Ağrı Training and Research Hospital of Turkiye in the year 2023, diagnosed with the VITEK‐2 Compact automated system (Biomérieux, France) and had antibiogram analysis conducted. Within the scope of the study, three strains of carbapenem‐resistant *A. baumannii*, two strains of carbapenem‐resistant *K. pneumoniae*, and three strains of intrinsic carbapenem‐resistant *S. maltophilia* were utilized.

This study was approved by the Scientific Research Ethics Committee of Ağrı İbrahim Çeçen University (Decision No: 459, Date: November 28, 2024) for the project titled “Determination of the Antibacterial Activity of Propolis Extracts Against Carbapenem‐Resistant Bacteria Isolated from Clinical Samples.”

### 4.7 Isolation of Clinical Strains

Blood, tracheal aspirate, sputum, and urine samples obtained from the Intensive Care Unit and Palliative Care Unit at Ağrı Training and Research Hospital were included in this study. In the Medical Microbiology Laboratory, blood cultures were incubated in the RENDER automated blood culture system (BC256, Blood Culture Systems, China). Other clinical samples were plated on 5% sheep blood agar (Oxoid, UK), eosine methylene blue agar (EMB, Oxoid), and, when necessary, chocolate agar (Merck, Germany), followed by incubation at 37°C for 24 h under aerobic conditions.

### 4.8 Antibacterial Susceptibility Testing

Using the VITEK‐2 Compact system, species‐level identification and antibiotic susceptibility tests were studied in line with the manufacturer's recommendations for *A. baumannii*, *K. pneumoniae*, and *S. maltophilia* strains. Carbapenem resistance of *A. baumannii* and *K. pneumoniae* strains were confirmed through the disk diffusion method (Bioanalyse, Türkiye). In addition, antimicrobial susceptibility of *S. maltophilia* strains were tested according to gradient test method for levofloxacin.

The strains were suspended in isotonic fluid to achieve a 0.5 McFarland turbidity standard. The isolates were inoculated onto Mueller–Hinton agar (MHA) (Oxoid) to perform antibiotic susceptibility testing. In addition, imipenem, meropenem, ertapenem discs and levofloxacin gradient tests (Bioanalyse, Türkiye) were placed on the agar. The plates were incubated for 18 ± 2 h at 35 ± 1°C with air. To assess the antibiotic susceptibility results, the European Committee on Antimicrobial Susceptibility Testing (EUCAST 2024 v.14.) guidelines were used. Only the breakpoint of levofloxacin was evaluated according to the Clinical and Laboratory Standards Institute (CLSI M100, 2023) for *S. maltophilia* strains. The quality control strain used *E. coli* ATCC 25922 and *P. aeruginosa* ATCC 27853.

### 4.9 Antimicrobial Activity Tests

Antimicrobial activity against clinical strains was determined using the well diffusion method [33]. For this purpose, fresh cultures of the strains were prepared using MHA medium. After preparing overnight the cultures, the samples were adjusted to a concentration of 0.5 McFarland using phosphate buffer saline (PBS). Using a cotton swab, samples at this concentration were inoculated onto MHA plates. After the plates dried, wells with a diameter of 6 mm were created, and 50 µL of tested propolis extracts' serial dilutions were added to the wells. After incubating the plates overnight at 37°C, the zone sizes of the wells were measured.

### 4.10 MIC and MBC

Fresh cultures of clinical samples found to be effective were prepared again and adjusted to 0.5 McFarland concentration. For this purpose, Müeller Hinton Broth‐II (MHB‐II) medium was used. For this purpose, 100 µL of MHB‐II medium was placed in each well of 90‐well U‐bottom plates. 100 µL of propolis samples prepared at a concentration of 10 mg/mL were added to the first wells and dilutions were made to other wells. Finally, 0.5 McFarland concentrations of the clinical samples were added to the relevant wells. Plates were left to incubate overnight at 37°C. The last concentration at which no growth was observed in the wells after incubation was determined as the MIC well. Before the MIC, 50 µL was taken from the wells and planted in MHA media, and the lowest value without growth was determined as MBC [33].

### 4.11 Statistical Analysis

Three sets of experiments were performed, and the acquired data underwent analysis using SPSS version 20.0 software (SPSS Inc., Chicago, IL, USA). To assess and compare the TPC, TFC, and FRAP, parameters across various species, statistical methods including one‐way ANOVA and Tukey's test were employed.

## Author Contributions

**Zeynep Ülkü Esertaş**: performed the experimental work. **Neşe Inal**: formal analyses. **Zehra Can**: performed experiments and drafted the manuscript. **Sevgi Kolaylı**: original manuscript writing and reviewing process handling.

## Conflicts of Interest

The authors declare no conflicts of interest.

## Data Availability

Data will be available on reasonable request.
